# A silver spoon effect reduces lifetime fitness in a declining loon population

**DOI:** 10.1007/s00442-025-05836-8

**Published:** 2025-11-24

**Authors:** Walter H. Piper, Claudia Kodsuntie, Hayden Walkush

**Affiliations:** 1 Schmid College of Science and Technology, Chapman University, Orange, CA 92866 USA; 2 College of Natural Resources, University of Wisconsin-Stevens Point, 800 N. Reserve Street, Stevens Point, WI 54481 USA

**Keywords:** Silver spoon, Carryover effect, Common loon, Population decline

## Abstract

A complete understanding of factors that influence animal fitness requires that we measure not only those occurring day to day in the life of an animal but also those that operate on longer time scales. Here, we investigated silver spoon effects (fitness impacts resulting from conditions faced early in life) and carryover effects (fitness impacts caused by environmental factors in a previous season) in a northern Wisconsin population of the common loon (*Gavia immer*). The mass of a loon chick divided by its age, an indication of food it received from its parents in its first 4 to 6 weeks of life (“chick condition”), affected both the likelihood of survival to adulthood and, among territory settlers, the number of chicks it fledged as an adult. Only one carryover effect was evident: increased ocean pH on the wintering ground had a modest positive effect on territory settlement rate. However, cohorts of loons that faced unfavorable ocean conditions in their first year yielded adults that fledged many chicks, which suggests that selection resulting from poor ocean conditions removed weaker phenotypes. The robust silver spoon effect in this species helps us understand a current and alarming pattern in the Wisconsin loon population: the sharp decline in the survival of chicks to breeding age.

## Introduction

Ecologists have known for some time that environmental conditions encountered by long-lived animals during one phase of life can affect their fitness profoundly in another (Greenberg and Marra 2005). In the wake of this realization, a holistic view of the lives of animals has begun to take hold: we cannot understand long-lived animals fully without considering multiple phases of the life history (Cooper et al. 2024).

Of course, the new, more expansive view of animal life histories presents daunting challenges. If we must track animals’ fitness across multiple life-history stages, we must also increase the scope of our research to encompass those stages. In addition, since many species move from one geographic area to another between seasons, we must, in some cases, study environments that are separated by several months and hundreds or thousands of kilometers. Fortunately, stable isotopes, telemetry, geolocators, or mere resightings of marked animals have permitted ecologists to link conditions that animals face during one season with fitness outcomes in later one (Ouwehand et al. 2016; Imlay et al. 2019; Cooper et al. 2024).

One pattern by which fitness impacts can span multiple life-history stages is the carryover effect. A carryover effect occurs when ecological conditions that an animal experiences in one life-history stage (such as the nonbreeding season) enhance or reduce its fitness during a subsequent stage (typically the breeding season; Harrison et al. 2011; Cooper et al. 2024). To date, difficulties following animals across seasons have limited the description of carryover effects to a handful of species (Harrison et al. 2011). A second cross-stage pattern, the “silver spoon effect”, occurs when individuals experience favorable conditions (e.g., more or higher quality food) in their first weeks or months of life relative to others raised in the same year or cohort and enjoy high fitness later in life (e.g., higher adult survival or breeding success) as a consequence (Grafen 1988; Van De Pol et al. 2006; Descamps et al. 2008; Hamel et al. 2009; Ancona and Drummond 2013; Mainwaring et al. 2023). Silver spoon effects have been found widely in animals (Albon et al. 1992; Vasilieva and Tchabovsky 2020; Sanghvi et al. 2021; Berzins et al. 2024).

Although most research on lagged effects such as carryover and silver spoon focuses on their impact at the level of the individual, both patterns influence population dynamics as well (Lindström 1999; Harrison et al. 2011; Berzins et al. 2024). Indeed, the potential impacts of lagged fitness effects on population dynamics constitute a new frontier in animal ecology.

We looked for impacts of lagged fitness effects on a population of common loons (*Gavia immer*) in northern Wisconsin that has been under continuous study since 1993. Common loons (hereafter “loons”) are large piscivorous diving birds well known for their nocturnal vocalizations. Routine capture and weighing of loons as chicks provided an opportunity to investigate possible silver spoon effects, because many banded chicks return as adults to the study area, settle on territories, and breed (Piper et al. 2012). Banding recoveries together with an investigation using satellite transmitters and geolocators have revealed that 80% of Wisconsin loons winter along Florida’s Gulf Coast (Kenow et al. 2021), which has made it possible to investigate carryover effects from winter to summer using long-term measurement of ocean attributes from that region.

We had an additional motive in looking for lagged effects on fitness in common loons. The loon population in northern Wisconsin has suffered a substantial decline, including an alarming increase in mortality of young adults (Piper et al. 2020). We sought to learn whether the massive die-off of young adults might result from a silver spoon effect, carryover effect(s) related to habitat degradation on the wintering grounds, or some combination of these factors.

## Methods

### Study area and study animal

From 1993 to 2024, we studied marked loons within a cluster of breeding territories covering an area of 2400 km^2^ in Oneida, Vilas, and Lincoln counties, Wisconsin (geographic center of study area: 45.7077° N, − 89.5930 W). Loon territories in this region comprise either entire small lakes (*N* = 130; mean size ± SD = 42 ± 34 ha), or portions of large lakes (*N* = 54 territories on 24 different lakes; mean size ± SD = 387 ± 403 ha). Agricultural activity is minimal in our study area, and most lakes with loons are bordered by hardwood and coniferous forest. Still, most study lakes have highly developed shorelines and experience constant recreational activity in the form of boating, angling, and swimming.

Adult loons show high site fidelity, tending to return to the same breeding territory year after year (Piper et al. 2023). Maintaining possession of a territory requires vigilance and active defense, however. Young adults (roughly 2 to 6 years of age) intrude constantly into breeding territories (Piper et al. 2006), preferring to settle on lakes of similar size and pH to their natal one (Piper et al. 2013). Settlement by young loons occurs by one of three means: 1) replacement of a dead pair member, 2) eviction of an existing pair member and pair formation with the mate of the displaced bird, or 3) founding of a new territory with a new mate (Piper et al. 2000). Territories that produced chicks the previous year are especially prone to intrusions and evictions, because loons use the presence of chicks as a cue indicating territory quality (Piper et al. 2006).

Loon chicks are semiprecocial and begin to take short dives within a few days of hatching. However, almost 100% of their food comes from parents through 5 weeks; by 8 weeks, they capture about 50% of their own food (Paruk et al. 2021). At 11 weeks, they provide 90% of their own food and have learned to fly (Paruk et al. 2021). During October and early November of their first year, most juveniles leave their natal lake and forage on others nearby that are: 1) of similar pH to their natal lake or 2) large and contain abundant food (Hoover et al. 2021).

Based on satellite tracking of juveniles (Kenow et al. 2021), we can now describe the fall migration of juveniles as follows. By mid-November, most juveniles have left the vicinity of their natal lakes and begun to travel south and east. They make brief stops on Lake Michigan; lakes in Iowa, Illinois, Indiana, Ohio or Missouri; and/or reservoirs in the southeastern U.S., before reaching the wintering grounds in the latter half of November.

Young loons from the Wisconsin population do not return to the breeding area until they reach 2 to 3 years of age (Piper et al. 2015). Instead, 1- and 2-year-olds spend their summers on the Atlantic coast, venturing as far north as the Canadian Maritime provinces (Kenow et al. 2021).

### Capture and sexing of chicks

We caught adult loons and their chicks of about 5 weeks (mean age 34.4 ± 6.6 d SD; *N* = 1109) by spotlighting and netting at night from a 4 m motorboat (Evers 1993). We transported captured loons to shore and weighed them with a digital scale (± 10 g; Brecknell SA3N340 ElectroSamson, Fairmont, Minnesota, USA). We then fitted adults and chicks 4 weeks and older with a numbered United States Geological Survey (USGS) band and three colored plastic leg bands (Gravoglas 2-plex; Gravotech, Duluth, Georgia, USA) in a unique combination. Following banding and weighing, family members were released together in their territories.

We determined the sex of 794 of 1109 total chicks in our sample. Of these, 613 were sexed through genetic analysis by a collaborator (A. McMillan, Buffalo State Univ; see Itoh et al. 2001), 128 by observing their behavior when they intruded into or settled on territories (180 loons), and 53 by both means. However, among chicks banded between 1993 and 2007, sex was only determined for those that returned as adults.

### Field observations

Following an initial visit in late April or early May during which we identified the breeding pair from leg bands, we made 60 min visits at no longer than 8-day intervals from early May through August 10 between 0500 and 1300 to each breeding territory. The number of breeding territories under study increased from 12 in 1993 to a mean of 56.5 from 1994 to 2004, 90.1 from 2005 to 2014, and 106.7 from 2015 to 2024. On each visit, we determined whether the pair was: 1) not breeding, 2) incubating eggs, or 3) raising chicks. We estimated the hatching date by interpolating between the first visit to a territory on which the pair was still incubating and the first visit on which we observed a chick. (Incubation of the one to two eggs laid by females lasts 4 weeks.) Chick age was estimated by the number of days between the estimated hatch date and the date of capture.

Territorial intrusions occurred commonly during our visits to loon territories, and intruders were often banded (e.g., 126 of 300 intruders in 2018; 42%). Our efforts to identify intruders from leg bands were crucial to our analysis of survival to adulthood, because 48% (*N* = 163) of all loons that we had banded as chicks and returned to Wisconsin (*N* = 337) were seen only as intruders between the age of 2 and 4 years and never settled on a breeding territory (Piper et al. 2015).

Intensity of field observation varied during the study and seemed likely to affect our probability of reobserving a loon banded as a chick on the breeding grounds. Based on the frequency with which young loons were observed in their first years on the breeding grounds (Piper et al. 2015), we therefore calculated observation intensity as a weighted index of the number of observations that occurred 2, 3, 4, and 5 years after the hatch year for each banded chick. Observation intensity was equal to 0.2 times the number of lake visits by all observers 2 years after the hatch year + 0.5 times the number of visits 3 years after hatch + 0.2 times visits 4 years post-hatch + 0.1 times visits 5 years post-hatch. For example, observation intensity equaled 1763 for chicks banded in 2013 but only 1087 for 2016 hatchlings owing to lower rates of field observation in 2018–2021 than from 2015 to 2018.

Natal dispersal by males (mean ± SD: 10.0 ± 6.7 km, *N* = 137) is, on average, of much shorter distance than that of females (32.0 ± 42.6, *N* = 60). Thus, many females that we banded as chicks survived to adulthood but were never resighted, because they settled outside of our study area. This pattern created a resighting bias that we accounted for by including sex as a covariate in our analysis of return rate.

### Measurement of ocean attributes

We do not know the exact wintering location of any loon banded in our Wisconsin study area. However, we know that 80% of our loons spend their winter months (late November through early April) along Florida’s Gulf Coast between Pensacola and the Florida Keys (Kenow et al. 2021). Wintering loons use estuaries and coves and also occur offshore as far as 120 km along the West Florida Continental Shelf (Kenow et al. 2021; eBird 2025).

A long-term database maintained by EPCHC (Environmental Protection Commission of Hillsborough County), near Tampa Bay, Florida, contains ocean data collected monthly, at consistent locations in the Tampa area, and by consistent methods since 1972. These data seemed appropriate to investigate the impact of ocean attributes on Wisconsin loons for three reasons. First, ocean properties seemed likely to influence prey availability, underwater foraging conditions, and/or health and survival of loons during winter. Second, based on recoveries of our loons that have died in winter months, Tampa appears to be a “hotspot” for Wisconsin loons. Ten of 34 Florida loon recoveries through 2022 occurred within 75 km of Tampa Bay (29%), a stretch that covers only 12% of Florida’s Gulf coast. Third, Tampa is the closest city to the midpoint of Florida’s Gulf Coast, roughly equidistant between the western edge of the range (the Alabama border) and the Keys to the south. Thus, ocean water at Tampa should provide an indication of ocean attributes of the Florida Gulf Coast as a whole.

Seawater measurements that we investigated for their potential influence on loon fitness were those taken monthly by EPCHC from November through March (loons’ wintering period) at “Station 94”, which was at Tampa Bay Buoy R10 in Egmont Channel. This point was far enough away from rivers and channels feeding Tampa Bay that its measurements reflect the coastal conditions that most Wisconsin loons face. The measurements we used were water clarity (secchi disk), temperature, dissolved oxygen, pH, salinity, ammonia, nitrates/nitrites, organic nitrogen, total phosphorus, and chlorophyll-*a* concentration. Field measurements of these ocean attributes followed the Florida Department of Environmental Protection 2017 Standard Operating Procedures for Field Activities DEP SOP001/01 (https://floridadep.gov/dear/quality-assurance/content/dep-sops). In situ measurements of temperature, salinity, pH, and dissolved oxygen were gathered by means of a multiprobe sonde (Aqua Troll, Hydrolab Quanta or Surveyor model). Secchi depth was measured ± 0.1 m. Water samples for nutrients and chlorophyll-*a* were collected using a beta sampler (Wildco® Beta™ Horizontal Water Bottle). Lab analysis occurred for chlorophyll-*a*, total phosphorus, and nitrates following Standard Method 10200 H 20th Ed., EPA 365.4/AQS400, and Standard Method SM4500NO3F/AQS400, respectively.

### Statistical analysis

#### Investigating survival, territory settlement, and reproductive success

We examined potential predictors of two binary variables and one continuous variable related to the fitness of young loons: 1) return or non-return to the breeding grounds; 2) settlement or not with a mate on a breeding territory; and 3) number of chicks reared to 5 weeks of age. We used natal territory as a random effect in each analysis to account for the fact that the 1109 loons in the sample were reared on only 163 different territories. We used generalized logistic mixed models (“xtlogit” command in Stata 18.5; Stata-Corp, College Station, TX, USA) to run the analyses of survival and settlement and a linear mixed model (“mixed” command in STATA) to run the analysis of the number of chicks.

#### Predictors examined and model selection

Sex was an especially important covariate examined as a potential predictor of fitness. Male loons disperse shorter distances from their natal lakes to breed than females (see above) and are therefore more often reobserved in the study area as young adults at 2 to 4 years of age. In addition, males tend to outweigh females as chicks. Thus, these two patterns raised the possibility of finding a spurious relationship between chick condition and return rate or one that appeared stronger than it actually was. Inclusion of sex as a covariate accounted for this potential bias. We also restricted our analysis of return rate to chicks hatched from 2008 to 2020, because we only determined sex from DNA in those years. Other known or suspected covariates of fitness that we tested were total observer-hours during the interval from 2 to 4 years after a chick’s banding, distance from a chick’s natal lake to the geometric center of the study area, and year.

We used a chick’s mass at capture divided by its age in days (“chick condition”, in units of g/day) as a potential predictor of fitness. In loons, mass is linearly related to age over the narrow 3- to 6-week-old age window during which we captured and weighed chicks, a pattern seen in other slow-developing birds (e.g., Huin and Prince 2000; Lok et al. 2014). Our measure of chick condition, if associated with fitness, would indicate a silver spoon effect.

Finally, we tested attributes of each loon’s natal lake and winter ocean attributes as potential predictors of fitness. Lake attributes included area, maximum depth, pH, and long-term water clarity. Attributes of ocean water during winter were based on measurements at Tampa Bay from EPCHC measurements. They comprised monthly measures of all attributes available throughout our period of investigation (1993 to 2020) from November through March: water clarity, temperature, dissolved oxygen, pH, salinity, ammonia, nitrates/nitrites, organic nitrogen, total phosphorous, and chlorophyll-*a* concentration.

We did not include cohort effects in our models. Animals in landscapes that experience widespread climatic or biotic fluctuations affecting many breeders simultaneously, such as colonial seabirds facing booms or crashes in fish populations (e.g., Erikstad et al. 2013) or deer born in dense populations and during cold springs (Kruuk et al. 1999), are prone to cohort effects (Beckerman et al. 2002). However, most of our loon pairs bred alone on small lakes (e.g., 84 of 105 pairs in 2020; 80%) or were one of two pairs on lakes of moderate size (14 pairs on 7 lakes; 13%). Hence, almost all chicks in our study were reared on small lakes with unique size, shape, geographic orientation, bathymetry, water chemistry, and biota. Thus, the peculiarities of the natal lake were probably more influential on rearing conditions, especially the kind and abundance of food chicks received from parents, than physical or biotic aspects of the larger landscape. In any event, our chick condition variable (mass/age) incorporated influences of the landscape, peculiarities of a chick’s natal lake, and degree of care shown by parents into a single variable that provided a good measure of each chick’s ability to thrive and grow in its first 4 to 6 weeks.

Model selection proceeded by testing predictors of return, settlement, and breeding success one by one, starting with covariates, and retaining those whose addition caused the Akaike information criterion (AIC) to decrease by ≥ 2. Models that differed by less than 2 in AIC were regarded as equivalent. For all three analyses, we ran models with and without the random effect and dropped it if it did not cause AIC to decline by 2 units or more.

#### Examining predictors of chick condition

Since we were chiefly interested in the possible existence of a silver spoon effect, we also explored predictors of chick condition (mass/age in g/day) itself. Chicks were reared on a single lake or part of a lake, so we limited our potential predictors to the following: year, lake size (natural log-transformed to normalize: maximum value 200 ha), maximum depth, pH (measured with a pH meter in August 2005), and July water clarity (mean of satellite measurements in July of a chick’s hatch year; see Piper et al. 2024). Again, we included natal territory as a random effect, because many chicks in our sample were reared on the same territory. We employed the “mixed” command in STATA for the analysis. Model selection proceeded as with the three fitness attributes.

## Results

### Predictors of juvenile return

**Fig. 1:**
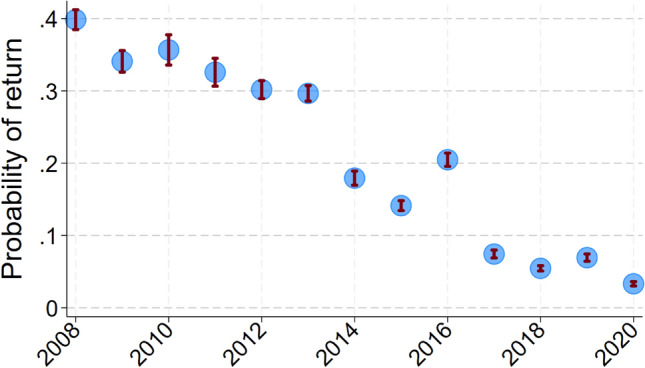
Mean predicted values by year (± SE) from the best-fitting model to determine the probability of return. Data were only available from 2008 on because sex was not determined for all chicks before 2008

**Fig. 2:**
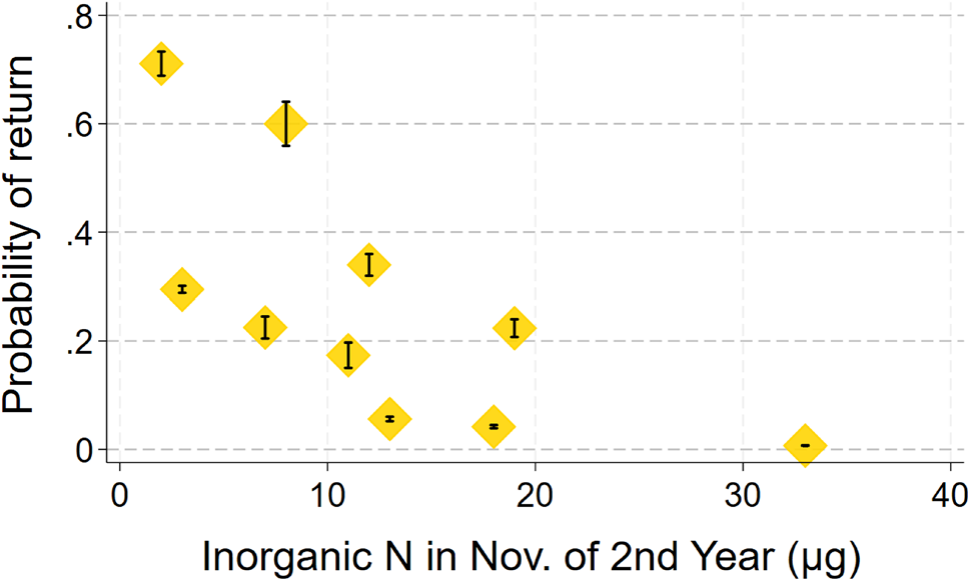
Mean predicted probability of return (± SE) by concentration of nitrates + nitrites in November of the second year of life

**Fig. 3:**
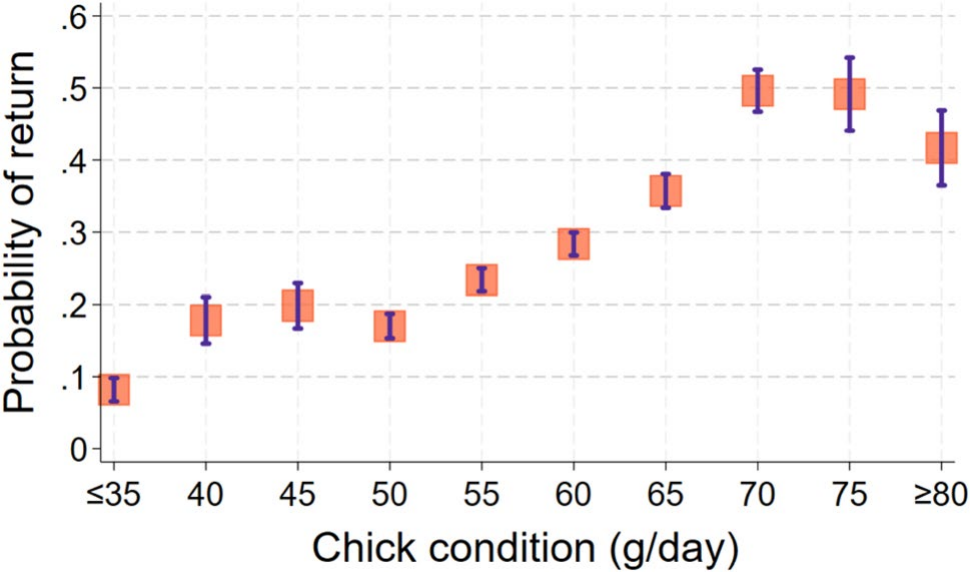
Mean predicted probability of return ± SE by chick condition

Inclusion of the random effect natal territory in models to predict the return of juveniles in adulthood caused AIC to increase by 2.0, so we dropped this term, which simplified the analysis to a logistic regression. Thus configured, a single model containing four predictors of juvenile return produced the smallest AIC value (Likelihood Ratio χ_5_^2^ = 71; *p* < 0.0005; *N* = 596 banded chicks from 136 lakes; Table 1), but two other models, each with five predictors, produced AIC values that exceeded that of the best model by only 0.77 and 1.80 (Table 1). Overall, the strongest predictor of return rate was year; return probability declined 13% each year (ΔAIC = + 7.1 when dropped from the top model; Fig. 1). The second strongest predictor of return was nitrate/nitrite concentration in November of the second year; return rate increased by 9% for µg/L decrease in second-year inorganic N concentration (ΔAIC = + 6.6 when dropped; Fig. 2). The third strongest predictor of return was the covariate sex; return rate increased in probability by 87% among males (ΔAIC = + 5.6 when sex was dropped). The fourth strongest predictor of return rate was chick condition; return rate increased by 2.7% for each increase in gram/day (ΔAIC = + 3.3 when dropped; Fig. 3). The predictors ocean clarity in December of the first winter and ocean clarity in March of the first winter each appeared in one of the top three models and were positively associated with return rate.

**Table 1:** List of logistic regression models to predict probability of return by loons banded as chicks by increasing AIC value

Cons	Ck cond	Sex	Year	Dclar1st	Mclar1st	Nnitr2nd	AIC	ΔAIC	Relative likelihood	Weight
X	+	+	−			−	542.89	0	1.00000	0.27956
X	+	+	−		+	−	543.67	0.774	0.67909	0.18691
X	+	+	−	+		−	544.69	1.795	0.40759	0.11096
X	+	+	−	+	+	−	545.5	2.61	0.27117	0.07294
X	+	+		+		−	545.6	2.704	0.25872	0.06959
X	+	+	−	+	+	−	545.67	2.771	0.25020	0.06720
X		+	−			−	546.18	3.287	0.19330	0.05184
X	+	+	−		+	−	546.67	3.776	0.15137	0.04057
X	+	+	−		+		547.4	4.507	0.10503	0.02814
X	+	+		+	+	−	547.59	4.7	0.09537	0.02554
X	+	+	−	+			547.66	4.769	0.09214	0.02468
X	+		−			−	548.13	5.233	0.07306	0.01957
X	+	+	−	+	+	−	548.65	5.756	0.05625	0.01506
X	+	+	−				549.34	6.443	0.03990	0.01068
X	+	+				−	549.97	7.072	0.02913	0.00780
X	+		−	+	+	−	551.23	8.339	0.01546	0.00414
X		+	−				553.28	10.381	0.00557	0.00149
X	+	+				−	553.3	10.401	0.00551	0.00148
X	+		−				555.02	12.13	0.00232	0.00062
X	+	+	−		+		555.52	12.625	0.00181	0.00049
X	+	+	−	+			557.19	14.291	0.00079	0.00021
X						−	563.88	20.984	0.00003	0.00001
X			−				564.4	21.504	0.00002	0.00001
X	+	+					590.39	47.496	0.00000	0.00000
X	+						594.94	52.049	0.00000	0.00000
X				+			605.31	62.419	0.00000	0.00000
X							606.16	63.267	0.00000	0.00000
X					+		607.34	64.442	0.00000	0.00000

Each row shows a different model, its associated AIC value, and the degree of support it received compared to all others tested

“+” indicates that predictor was positively associated with return probability, “−” indicates a negative association. Useful predictors included year, sex, chick condition (mass/age), water clarity in December and March of the first winter, and nitrate/nitrite concentration in November of the 2nd winter

### Predictors of territory settlement

Only two predictors were associated with successful settlement on a territory: sex and ocean pH in March of the first winter (Likelihood ratio χ_2_^2^ for best model = 21.0; *p* < 0.0005; *N* = 135 returning chicks from 77 lakes). Again, inclusion of natal territory as a random effect caused AIC to increase by 2.0, so we dropped it and simplified the model to a logistic regression. Males were 5.4 times more likely to settle on territories than females (ΔAIC = + 16.6 when dropped). Likelihood of settlement increased by 5.2% for each 0.1 unit increase in pH in March of a loon’s first winter (ΔAIC = + 2.0 when dropped).

### Predictors of chicks fledged

**Fig. 4:**
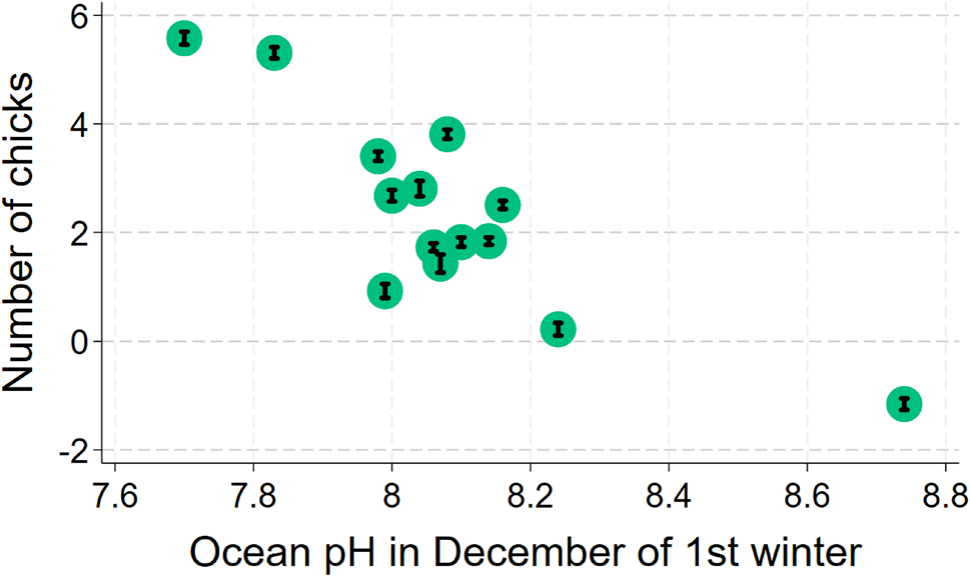
Means ± SEs of predicted values for number of fledged chicks by ocean pH in December of first winter of life. (Since these are predicted values from the model, some fall outside of the possible range.)

**Fig. 5:**
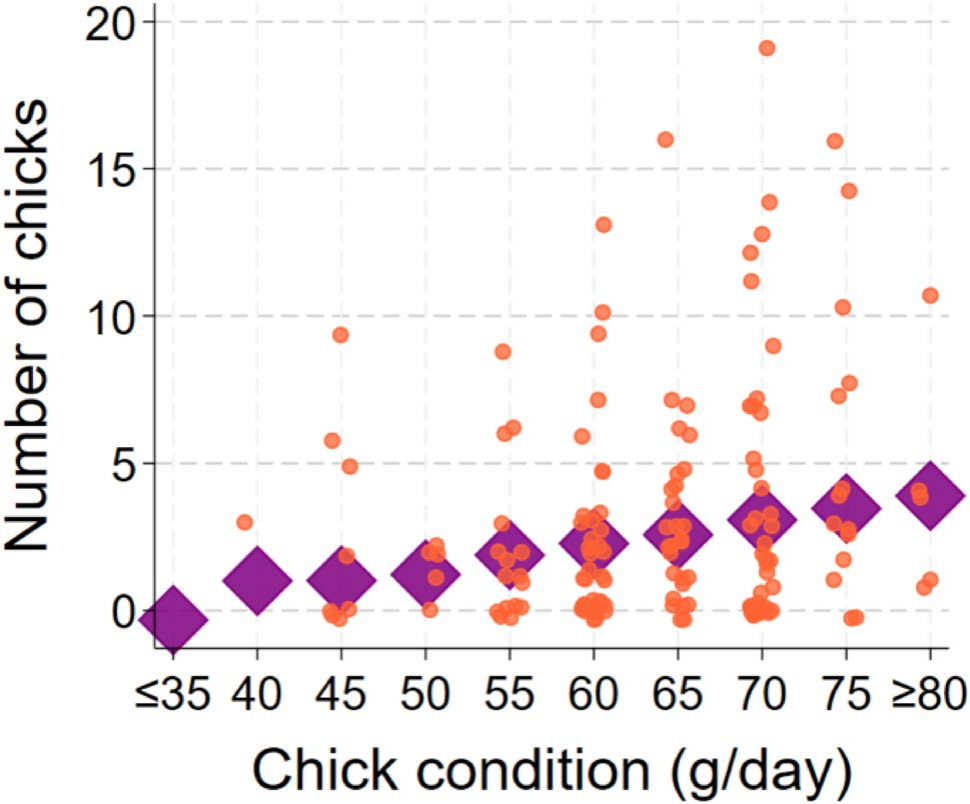
Means of predicted values of chicks (diamonds) and distribution of actual numbers of chicks by chick condition for loons that settled on territories

Among settlers, six predictors were associated with the number of chicks a loon fledged during its lifetime (Best Model: F_6_^89^ = 3.84; *p* < 0.0019; *N* = 96 chicks from 62 territories that returned and settled; Table 2). However, a second model predicted chick production almost as well (ΔAIC = + 0.13; Table 2). Again, AIC fell by 2.0 when the natal territory was dropped as a random effect, so we ran a multiple regression analysis. Ocean pH in December of the first year was the factor associated most strongly with lifetime chick production. The number of chicks increased by 0.79 for each 0.1 unit decrease in December ocean pH in the first winter (ΔAIC = + 7.4 when dropped; Fig. 4). Second, organic nitrogen in November of the first year was negatively associated with chick production; loons produced 1.0 more chicks for every 0.1 mg/L decrease in the concentration of organic nitrogen in seawater (ΔAIC = + 6.1 when dropped). Third, chick production increased by 0.9 per meter decrease in water clarity in January of the first winter (ΔAIC = + 3.6 when dropped). Fourth, the number of chicks fledged increased by 0.07 per unit increase in chick condition (g/day; ΔAIC = + 2.5 when dropped; Fig. 5). Fifth, chick production rose by 0.13 chicks for every 1 µg/L increase in inorganic nitrogen in December of the first winter (ΔAIC = + 2.2 when dropped). Finally, inorganic nitrogen in January of the second winter showed a possible negative association with chick production. The predictor appeared in only three of the eight models within 4 AIC units of the top model. The number of fledged chicks rose by 0.11 for each 1 µg/L decrease in inorganic nitrogen in January of the second winter (ΔAIC = + 0.13 when dropped).

**Table 2:** List of regression models to predict number of chicks fledged by loons that were banded as chicks

Cons	Ck cond	DNitr1st	NOrgN1st	Jclar1st	DpH1st	JNitr2nd	AIC	ΔAIC	Relative likelihood	Weight
X	+	+	−	−	−	−	490.01	0	1	0.2558
X	+	+	−	−	−		490.14	0.129	0.937536	0.2398
X			−	−	−		491.8	1.793	0.407995	0.1044
X	+		−	−	−	−	492.23	2.224	0.328901	0.0841
X	+		−	−	−		492.31	2.3	0.316637	0.081
X		+	−	−	−	−	492.53	2.527	0.282663	0.0723
X	+	+	−		−		493.61	3.599	0.165382	0.0423
X			−	−	−		493.66	3.655	0.160815	0.0411
X	+	+	−		−	−	494.53	4.525	0.10409	0.0266
X	+		−		−		494.66	4.656	0.097491	0.0249
X	+	+		−	−	−	496.14	6.128	0.046701	0.0119
X					−		497.19	7.187	0.027502	0.007
X	+	+	−	−		−	497.4	7.39	0.024847	0.0064
X				−			499.54	9.534	0.008506	0.0022
X	+						499.65	9.639	0.008071	0.0021
X							500.13	10.126	0.006327	0.0016
X						−	500.95	10.939	0.004213	0.0011
X		+					501.26	11.257	0.003594	0.0009
X			−				501.35	11.344	0.003441	0.0009

Each row shows a different model, its associated AIC value, and the degree of support it received compared to all others tested

“+” indicates that predictor was positively associated with return probability, “−” indicates a negative association. Among predictors that showed at least a weak association with chick production were ocean pH in December of the first year, organic nitrogen in November of the first year, water clarity in January of the first winter, chick condition, inorganic nitrogen in December of the first winter and inorganic nitrogen in January of the second winter

### Predictors of chick condition

**Fig. 6:**
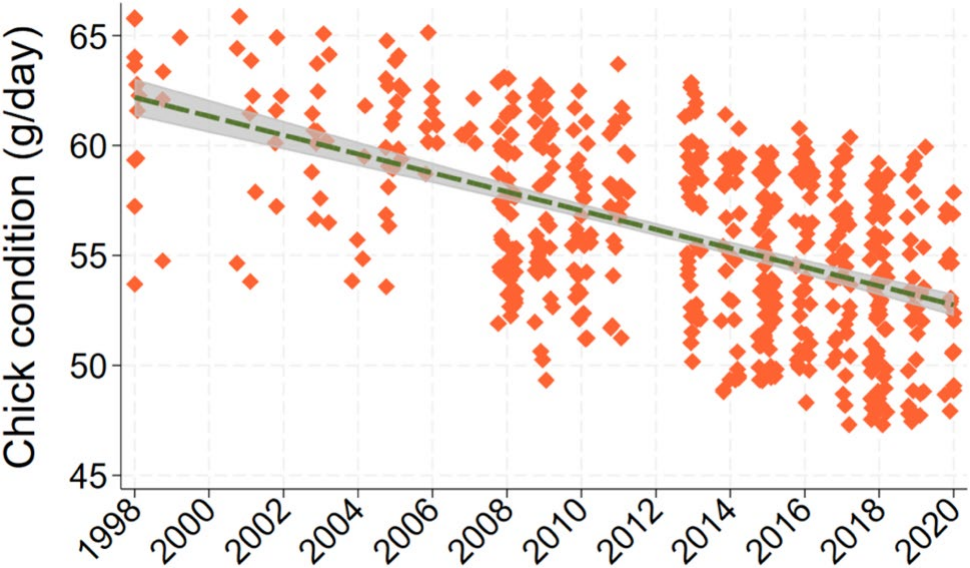
All predicted values for chick condition by year. Line shows the slope of the relationship and its 95% confidence interval

**Fig. 7:**
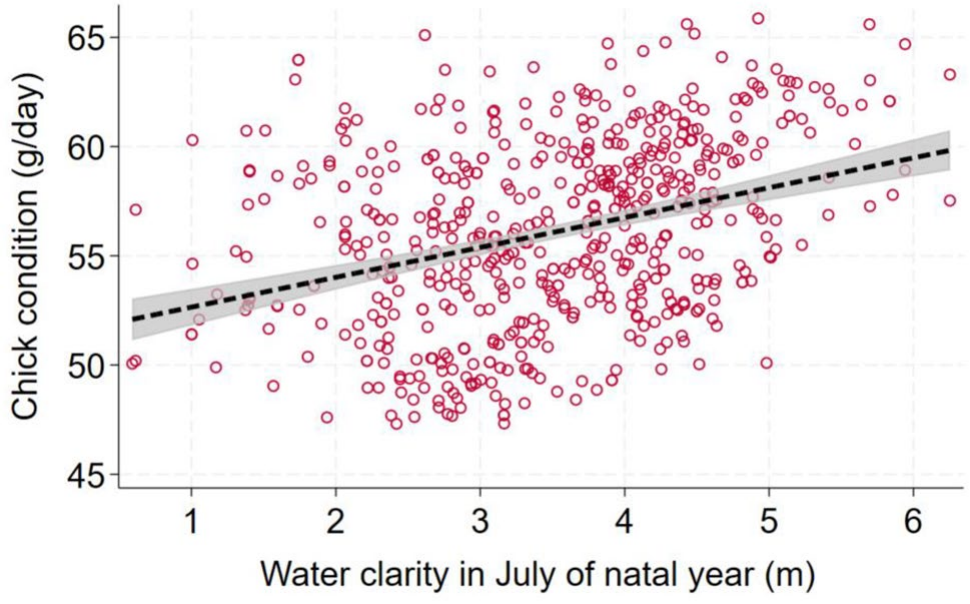
All predicted values of chick condition for loons plotted against mean July water clarity in their natal year. Line shows the slope of the relationship and its 95% confidence interval

**Fig. 8:**
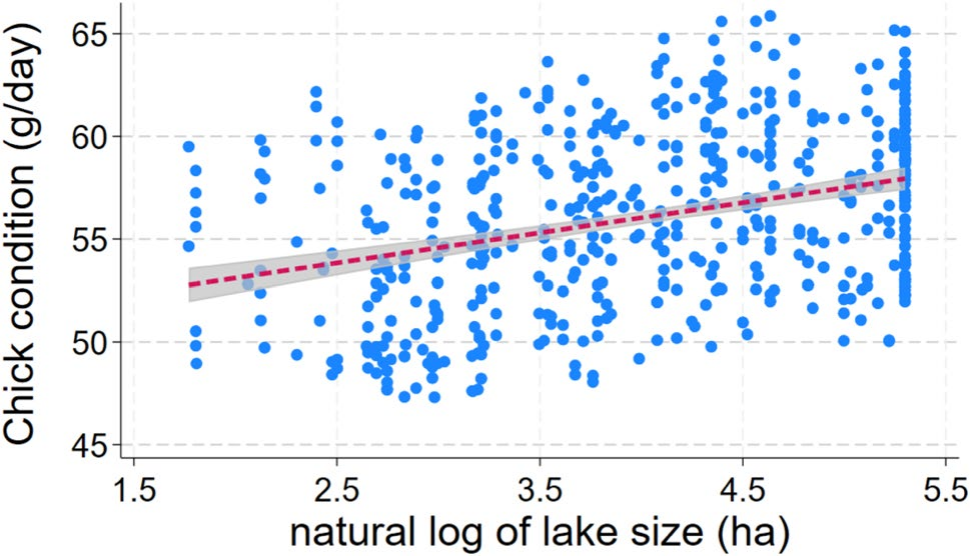
All predicted values for chick condition by natural logarithm of lake size. Line shows slope of the relationship and its 95% confidence interval

Nine models fell within 2 AIC of the best one in the analysis of chick condition (F_6_^555^ for best model = 17.1; *p* < 0.0005; *N* = 562 chicks from 128 territories; Table 3). Sex, a covariate, was the most important predictor; condition was 5.8 g/day higher among males (ΔAIC = + 46.9 when dropped). Year was the second most important predictor of chick condition, which has declined steadily during the study period at a mean rate of 0.31 g/day a year (ΔAIC = + 13.6 when dropped; Fig. 6). Water clarity influenced condition in two respects. Condition increased by 0.97 g/day for each 1 m increase in mean water clarity in July during the natal year (ΔAIC = + 1.7 when dropped; Fig. 7), but decreased by 0.38 g/day per meter increase in long-term mean clarity (ΔAIC = + 0.5 when dropped). Lake size was the next most important predictor; condition increased by 0.92 g/day for each 1 natural log unit increase in lake size (ΔAIC = + 1.6 when dropped; Fig. 8). Maximum lake depth was possibly important, as condition rose by 0.19 g/day for each meter increase in maximum depth (ΔAIC = + 0.7 when dropped). Lake pH showed a weak association with condition, which increased by 1.6 g/day for each unit increase in pH (ΔAIC = + 1.4 when dropped).

**Table 3:** List of regression models to predict chick condition (g/day) of loons that were banded as chicks

Const	Sex	Year	Lnlksize	LTclar	Maxdep	Julyclar	pH	AIC	ΔAIC	Relative likelihood	Weight
X	+	−	+	−	+	+		4165.706	0.000	1.000	0.148
X	+	−		−	+	+	+	4165.875	0.169	0.919	0.141
X	+	−	+	−		+		4166.434	0.728	0.695	0.104
X	+	−	+	−		+	+	4167.029	1.323	0.516	0.076
X	+	−	+	−	+	+	+	4167.036	1.330	0.514	0.076
X	+	−		−	+	+		4167.261	1.555	0.460	0.068
X	+	−	+			+	+	4167.483	1.777	0.411	0.061
X	+	−		−	+		+	4167.562	1.856	0.395	0.058
X	+	−	+	−	+			4167.574	1.868	0.393	0.058
X	+	−	+				+	4167.973	2.267	0.322	0.048
X	+	−	+					4168.598	2.892	0.236	0.035
X	+	−	+			+		4168.784	3.078	0.215	0.032
X	+	−		−	+			4169.189	3.483	0.175	0.026
X	+	−	+	−				4169.580	3.874	0.144	0.021
X	+	−	+		+			4169.660	3.954	0.138	0.020
X	+	−	+	−			+	4169.753	4.047	0.132	0.020
X	+	−	+		+	+		4170.612	4.906	0.086	0.013
X	+	−			+			4176.369	10.663	0.005	0.001
X	+	−						4177.626	11.920	0.003	0.000
X	+		+	−	+	+		4179.314	13.608	0.001	0.000
X	+							4197.857	32.151	0.000	0.000
X		−						4224.480	58.774	0.000	0.000
X				−		+		4228.315	62.609	0.000	0.000
X							+	4241.149	75.443	0.000	0.000
X						+		4241.319	75.613	0.000	0.000
X			+					4241.385	75.679	0.000	0.000
X				−				4248.323	82.617	0.000	0.000
X								4249.265	83.559	0.000	0.000
X					+			4249.613	83.907	0.000	0.000

Each row shows a different model, its associated AIC value, and the degree of support it received compared to all others tested

“+” indicates that predictor was positively associated with return probability, “−” indicates a negative association. Potential predictors included year, and five attributes of the natal lake: lake size, short- and long-term water clarity, maximum depth, and pH

## Discussion

### Effects of winter ocean conditions

Our long-term study of fitness in a breeding population of common loons has made one pattern clear. Physical and chemical attributes of the lakes in which the birds were raised and the ocean in which they spent their winters affect their survival and breeding success throughout life.

It is perhaps surprising that we could detect effects of winter ocean attributes on loon fitness at all. Eighty percent of loons breed in northern Wisconsin winter along Florida’s entire Gulf coastline, from Pensacola to the Keys, a length of 1239 km, and the remaining 20% in winter along the Atlantic coast from southern Florida up to the Carolinas (see also Kenow et al. 2021). We used ocean measurements from only one point within this extensive range. The fact that we detected significant effects despite this limitation suggests that: 1) ocean conditions in Tampa might be representative of conditions along the entire Florida Gulf Coast (and perhaps, to an extent, the Atlantic coast), and/or 2) a large enough proportion of our loon population winters near Tampa Bay that patterns were detected. However, the limited precision with which we measured ocean attributes for our wintering population as a whole requires us to interpret our findings with caution.

The concentration of inorganic nitrogen (e.g., NH_4_^+^ and NO_3_^−^) in ocean water in a loon’s second winter was negatively associated with return rate to the breeding grounds as an adult. This was, in fact, the only second-year oceanic factor that we found to affect loon fitness strongly. Inorganic nitrogen increases are known to fuel the growth of phytoplankton, so it seems likely that high algal growth spawned by inorganic nitrogen reduced survival among second-year loons, which in turn produced a lower return rate. Of course, it is curious that this effect occurred in the second year of loons’ lives, not the first, when loons would seem more vulnerable to environmental hazards. In addition, pH in March of the first winter had a weak positive association with the settlement rate of young adults. Since higher ocean pH promotes survival in a variety of marine fishes, crustaceans, and mollusks (Kroeker et al. 2013), this result likely means that a favorable foraging environment allows loons to remain in good condition, producing a positive carryover effect on settlement (which occurs at 4 to 8 years; Piper et al. 2015).

While favorable ocean conditions appeared to promote high survival and settlement, poor ocean conditions were associated with high chick production. The strongest predictor of chick production was low ocean pH in a loon’s first December, which generally reduces the abundance of marine animals (Kroeker et al. 2013). Fledging of chicks among adult loons was also predicted by two other first-year patterns: high inorganic nitrogen in December and low water clarity in January, both of which are likely indications of phytoplankton growth (e.g., algal blooms) and consequent poor foraging conditions (Regnier and Steefel 1999; Abdelrhman 2016). The relationship between chick production and low organic nitrogen (e.g., amino acids, proteins) in the first November too implies that unfavorable, unproductive ocean conditions early in life were associated with more chicks being fledged (Letscher et al. 2013), although some studies have found that organic nitrogen can support algal growth (Berman and Bronk 2003). In sum, low water quality during a yearly cohort’s first winter was consistently predictive of enhanced success in the fledging of chicks by that cohort.

The association between poor ocean conditions and high chick production is, on its face, difficult to explain. However, unfavorable conditions faced by first-winter loons likely resulted in natural selection that we did not detect in our measurements of return rate or territory settlement. If selection “weeded out” less fit competitors within yearly cohorts, then survivors within those cohorts were individuals of high fitness, which were likely to produce many chicks. In any event, several studies have reported that harsh conditions that a yearly cohort faces early in life can cause high mortality among poor phenotypes and thus leave behind a set of individuals of high fitness (Rose et al. 1998; Garratt et al. 2015; Drake et al. 2025).

But why did our measures of return rate and territory settlement not show the results of selection? There are two possible reasons for this oddity. First, the number of chicks fledged is a quantitative measure and is therefore likely to capture the variability in fitness better than the binary variables used to measure return rate and settlement. Second, the number of chicks fledged during a loon’s lifetime is a more comprehensive measure of fitness than return or settlement, because it incorporates both long-term adult survival and the capacity to raise young successfully.

### Silver spoon effect

While we obtained little evidence of carryover effects in loons, a silver spoon effect was evident in two separate analyses: survival to breeding age and lifetime reproductive success. In fact, the silver spoon effect in loons, which affects the survival of young adults and the number of chicks they fledge, contrasts with most others reported in vertebrates, which are weak (Oort and Otter 2005), affect only one component of fitness or one sex (Tilgar et al. 2010; Douhard et al. 2014), occur during a narrow phase of the lifespan (Ancona and Drummond 2013; Hamel et al. 2016), or are expressed only under narrow environmental conditions (Pigeon et al. 2019; but see Song et al. 2019).

Of course, the prominent silver spoon effect in loons shines a spotlight on chick-rearing conditions. Qualities of a loon’s natal lake such as size, clarity, and depth strongly affect the amount of food it receives from its parents and, hence, its fitness throughout life. In general, loon chicks reared on large, deep lakes achieve better condition than those on small shallow ones. However, water clarity had differing impacts in the short and long term. While clear water during the month of July (when typical chicks are growing from 2 to 6 weeks of age) is favorable to chick growth, high long-term clarity of the natal lake was associated with reduced growth, suggesting that foraging efficiency of loon parents on very clear lakes might be sensitive to fluctuations in clarity (see also Piper et al. 2024).

Loons’ vulnerability to a negative silver spoon effect helps us solve a puzzle we encountered in our investigation of the Wisconsin population. Significant declines in survival of young and old chicks, chick mass, and brood size were accompanied by an alarming loss of young adults prior to territory settlement (Piper et al. 2020). It is now evident that the same poor breeding conditions that harmed chicks in the short term weakened them in the long term, causing high mortality and low lifetime breeding success among those that survived past fledging age.

Although the silver spoon effect provides a partial solution to the puzzle of the die-off of young adult loons, the very strong yearly declines in return rate and chick production (see Tables 1, 3) persist even after chick condition is considered. We detected these declines not as yearly cohort effects, which can fluctuate upwards and downwards over a span of many years, but as simple linear monotonic decreases in return rate and chick production. There seem to be two possible explanations for the strong downward trends in these fitness measures. First, our measurement of chick mass decline, based on a single mass measurement, might have failed to measure chick condition fully. If so, then the year predictor might have picked up some of the variability in chick mass lost in our single measurement. Second, an unmeasured environmental factor or factors, such as steadily increasing human boating activity, might be causing these two declines in loon fitness.

Our results suggest that silver spoon effects experienced by a loon in the first weeks of life on its natal lake are stronger than carryover effects encountered during its initial winter. Such a pattern would not be surprising. Yet, it is possible that carryover effects in loons are stronger than we have reported here. Our use of measurements from a single location probably represented ocean conditions poorly for many wintering loons in our breeding population. If we had data from ocean conditions across the winter range and could assess fitness impacts on individuals based on the ocean conditions they faced at their exact wintering location, we might have found more substantial carryover effects.

The potential consequences of silver spoon effects in animals generally seem underappreciated for two reasons. First, owing to the paucity of field studies with the bandwidth to mark and take measurements on animals during the developmental stage and collect fitness data on those same individuals as adults, we have only a smattering of reports of silver spoon effects. Yet, the taxonomic breadth of such effects implies that they occur in most animal species. Second, silver spoon effects are by nature cryptic; that is, they produce many individuals that appear normal in one life-history phase but in reality have low fitness that contributes to population decline. Thus, studies that rely solely upon measures of breeding success like lower rates of young fledging or reaching independence are likely to greatly underestimate the negative impact of poor developmental conditions on animal populations.

A robust silver spoon effect confers both good and bad news for ecologists studying threatened species. Traditionally, identifying the cause(s) of population decline has required careful study of multiple phases of the life history (Baillie 1990; Buehler et al. 2008). But if an animal’s condition in its first few weeks provides a good assay of its fitness throughout life, then conservation efforts aimed at improving rearing conditions during that brief, sensitive window might boost populations in the long term. On the other hand, few studies have the scope to measure silver spoon effects, so the potential to learn about the long-term fitness of animal populations through data collection early in their lives is largely unrealized.

We generally have a poor idea of how species extinctions occur (Cahill et al. 2013). Thus, another important outcome of our findings is the demonstration of an apparent link between low foraging efficiency and population decline. Many loons do not die quickly from starvation; rather, limited food received during the chick phase leads to phenotypically weak individuals that neither survive nor reproduce well. And therein lies the most troubling aspect of a negative silver spoon effect. Unfavorable breeding conditions produce young of poor quality, which may enter the adult population normally yet suffer downstream impacts such as low adult survival and/or low reproductive success (Albon et al. 1987). If sustained and population-wide, silver spoon effects can lead to long-term downturns in populations that show no obvious signs of decline (Albon et al. 1992).

## Data Availability

The datasets generated during and/or analyzed during the current study are available in the “Loon Project Database” repository through Chapman University Digital Commons (https://digitalcommons.chapman.edu/sees_data/3/).
